# A Modified Reach-to-Grasp Task in a Supine Position Shows Coordination Between Elbow and Hand Movements After Stroke

**DOI:** 10.3389/fneur.2019.00408

**Published:** 2019-05-08

**Authors:** Kate Broome, Irene Hudson, Kaitlyn Potter, Jason Kulk, Ashlee Dunn, Jameen Arm, Tom Zeffiro, Gavin Cooper, Huiqiao Tian, Paulette van Vliet

**Affiliations:** ^1^School of Health Sciences, University of Newcastle, Callaghan, NSW, Australia; ^2^Department of Mathematical Sciences, College of Science, Engineering and Health, Royal Melbourne Institute of Technology, Melbourne, VIC, Australia; ^3^School of Mathematical and Physical Sciences, University of Newcastle, Callaghan, NSW, Australia; ^4^Calvary Mater Hospital, Newcastle, NSW, Australia; ^5^Department of Diagnostic Radiology and Nuclear Medicine, University of Maryland Medical Center, Baltimore, MD, United States; ^6^Centre for Translational Neuroscience and Mental Health, University of Newcastle, Callaghan, NSW, Australia; ^7^Research and Innovation Division, University of Newcastle, Callaghan, NSW, Australia

**Keywords:** stroke, upper limb, reaching, coordination, grasp

## Abstract

**Objective:** A modified reach-to-grasp task has been developed for the purpose of investigating arm-hand coordination in a supine position in the functional magnetic resonance imaging environment. The objective of this study was to investigate the kinematics of the reach-to-grasp task, in stroke and healthy participants.

**Design:** Observational cohort study.

**Setting:** Movement laboratory.

**Participants:** Ten stroke participants and 10 age-matched healthy participants performed 10 repetitions of the modified reach-to-grasp task in two conditions—a natural condition and a standardized condition in a splint.

**Intervention:** Not applicable.

**Main Outcome Measures:** Kinematic variables of start time of transport, start time of aperture, movement duration, time of peak velocity (PV), percentage time of PV, peak deceleration (PD), percentage time of PD, peak aperture (PA), time of PA, and percentage time of PA were recorded. The correlation between key events in the grasp and transport trajectories were investigated. Performance between conditions and groups were compared.

**Results:** Both groups demonstrated a significant correlation between the start time of aperture and the start time of transport and between the time of PA and PV in both conditions. A significant correlation was found between the time of PA and the PD in both conditions for the healthy group, but in neither condition for the stroke group. Movements by participants with stroke had a significantly longer movement duration, a smaller PV, and an earlier absolute time of PV and PD, and an earlier percentage time of PV and PD. They also had a smaller aperture than healthy participants. Wearing the splint resulted in a significantly higher PV, later absolute and percentage time of PV, PD, and PA, and a smaller PA compared to moving without the splint. The timing of transport variables time to peak velocity and time to peak deceleration, were strongest determinants of movement duration.

**Conclusion:** The modified reach-to-grasp movement performed without the constraint of the splint, demonstrates similar motor control and coordination between the grasp and transport components of reach-to-grasp as in seated reach-to-grasp. This provides a new task that may be used to explore reach-to-grasp in the fMRI environment.

## Introduction

Approximately 77% of people with stroke experience impaired coordination of their upper limb (1). Coordination is defined as an ability to maintain a context-dependent and phase-dependent cyclical relationship between different body segments or joints in both spatial and temporal domains (2). A key functional movement of the upper limb is the ability to reach and grasp an object, therefore how this ability is affected following stroke is of great interest. Investigations of reach-to-grasp typically study reaching movements performed in a sitting position, however sitting does not suit all research environments, such as for example the fMRI environment. The primary objective of this study was therefore to investigate the kinematics of a reach-to-grasp task, performed in supine, in a group of people with stroke and a group of healthy individuals.

The kinematics of reach to grasp include a transport of the hand phase describing the movement of the hand toward the object, driven by extrinsic factors such as the location of the object (3, 4), and a grasp component describing the shaping of the hand to reflect the intrinsic factors such as size and shape of the object to be picked up (3, 4).

These two independent components of the reach-to-grasp movement must be coordinated to effectively grasp an object (5). Evidence of an invariant temporal relationship between these components has been demonstrated in the seated reach-to-grasp movement (5, 6) in healthy people. The initiation of grasp aperture is correlated with the transport component, occurring between 0.2 and 0.4 s of the beginning of the transport phase (5, 7). The peak velocity of the hand occurs within the first half of the movement duration, and the peak hand opening and peak deceleration of the hand occur in the second half of the movement duration, with peak hand opening preceding peak deceleration. There is a correlation between the time of peak hand opening and the time of peak deceleration (7) and between the time of peak hand opening and the time of peak velocity (7, 8). It is suggested these correlations ensure a coordinated representation stored in memory referred to as motor schema, for reach-to-grasp (5).

Substantive data from both computer simulations and real individuals support the idea that reach-to-grasp is controlled with respect to the expected movement duration to the target via a consistent hand closure time (6, 9, 10). There is a two-way interaction between the neural processes controlling transport and grasp, so that the expected duration to the target of each of these trajectories is compared and adjusted, so that they are temporally matched (6). For example, when both object size and location are “perturbed” at movement onset, peak aperture (PA) and peak deceleration (PD) are both delayed to allow them to be temporally matched again after adjustment to each component has been made (11). The expected duration to the target may be determined by a motor schema (12). Alternatively, the expected duration may be derived from internal models (13), acquired and stored in part by the cerebellum.

Motor impairment resulting from a stroke has a significant impact on the co-ordination of a reach-to-grasp movement. Previous research has found common trends including longer movement durations (14, 15), longer deceleration phase (7), abnormal timing of grasp (16) such as an earlier peak aperture (14, 17, 18) and delayed initiation of grasp aperture until the deceleration phase of transport (19), disruption of interjoint coordination (20), increased variability of peak aperture size (7, 18), and deficits in accuracy of transport and grasp (18). It has been reported that some stroke patients can reach directly to a target with relatively normal movement speed, but fail to shape their hand accurately for grasping (15, 18), while other patients reach to an incorrect location, even though they can form a proper grasp (21, 22).

A brief description of the brain activity subserving these kinematics is given here, though longer summaries are available (4). Different areas of the cortex are activated when performing either grasp, transport, or both components together. During the grasp phase, activation of the bilateral anterior intraparietal sulcus (aIPS) (23, 24) and the ventral premotor cortex (vPM area 6), occurs (24). For transport, the superior parieto-occipital cortex (24), the left rostral superior parietal lobe (area 5L), the dorsal premotor area 6 (24) and the medial intraparietal sulcus, and the precuneus (medial aspect of parietal lobe) (25) are activated. When the transport and grasp components are combined, the dorsal premotor cortex (PMd), supplementary motor area (SMA), and the somatomotor areas (S1, S2, and M1) are activated (24). There are also two parietofrontal neural circuits dedicated to determination of object location for hand transport (superior parietal lobule to dorsal premotor area 6) (26) and object size and shape for hand shaping (inferior parietal lobule to ventral premotor area 6) (27).

Much of our understanding of the effect of stroke on the motor control of reach-to-grasp has come from studying the motor performance of people with lesions in specific brain areas. For example, lesions in the parietal cortex result in delayed hand opening (28) and if the lateral bank of the anterior intraparietal area in the posterior parietal cortex is involved, in poor pre-shaping of the hand (29). Cerebellar lesions cause greater variability in the velocity profile, and more velocity peaks compared to healthy people (30) and grasp shows a larger and earlier peak aperture expressed as a percentage of movement duration (30) compared to healthy subjects and these deficits are accentuated in fast movements (31). People with lesions in the posterior limb of internal capsule demonstrate longer movement duration, later time to peak velocity, and a longer deceleration time (32).

However, neuroimaging studies in people with stroke using measures of brain activity such as fMRI *during* performance of meaningful movement tasks are sparse, being mostly limited to movements such as finger tapping or button pressing. fMRI studies involving reach-to-grasp movements would help to quantify the extent to which a lesion interrupts expected pathways and/or functional networks in individuals or distinct groups of patients. Analysis of the impact of the lesion on brain networks as well as knowledge of viable brain networks has potential to guide rehabilitation clinicians in stroke rehabilitation (4). The lesion may be mapped relative to functional brain regions and networks that are known to be important for particular functions and tasks. There would then be potential to individually tailor treatment according to the underlying neurobiology (33).

Practical problems associated with performing reach-to-grasp movements whilst in the fMRI environment have resulted in limited fMRI studies of arm movements related to everyday function, in people after stroke. The usual seated reach-to-grasp experimental paradigms cannot be applied, so these movements must be adapted to be performed in supine. One concern is that reach-to-grasp movements can result in associated head motion, which potentially creates noise that may be spuriously correlated with the fMRI signal [the blood-oxygenation-level dependent (BOLD) signal]. In order to distinguish these head motion artifacts from the fMRI signal, corrective algorithms must be applied to the data (34). To a certain extent, head motion can be prevented by instructions and training given to the participant, or by restraints such as vacuum cushions that fit inside the head coil. However, these methods are no guarantee for prevention of head motion. A reach-to-grasp movement that can be performed in the fMRI scanner with minimal head motion is therefore desirable.

Where the goal is to understand functional brain networks involved in the control of reach-to-grasp, it is also important that the task shows similar characteristics to tasks performed in everyday life, such as the seated reach-to-grasp task. Bearing these factors in mind, we have developed a modified reach-to-grasp movement that can be performed within the fMRI environment. In developing this test movement, an important consideration was whether task performance should be standardized by a splint, to ensure consistency of movement amplitude between trials and individuals as much as possible, or whether the movement should be performed naturally using visual cues to guide amplitude, which is more ecologically valid. The splint introduces the adverse possibility of altering the kinematic characteristics of the natural movement and so it would be useful to compare the kinematic characteristics during a splint and a natural condition.

The specific hypotheses to be tested in this study were that, as in the seated reach-to-grasp task, first: (a) the start time of hand opening would be correlated with the start of hand transport toward the object; (b) the time of maximum hand opening would be correlated with the time of peak deceleration of the hand; and (c) the time of maximum hand opening would be correlated with the time of peak velocity of the hand. Second, it was hypothesized that use of a splint to guide movement would cause significant changes to performance compared to movement without the splint. Third, people with stroke were expected to be significantly different to that of healthy individuals, with a longer movement duration, smaller peak velocity and peak aperture, and later time of peak velocity, peak deceleration and peak aperture. Fourth it was expected that the degree to which the timing of these variables covaried with movement duration, would be decreased in people with stroke compared to healthy individuals.

## Methods

### Participants

Ten participants with stroke were recruited consecutively according to the following inclusion criteria: (1) A clinically confirmed diagnosis of ischemic or haemorrhagic stroke; (2) Remaining upper limb movement deficits, i.e., Scoring < 75 on Wolf Motor Function test; (3) Reside within 50 km of the laboratory; and (4) Give informed written consent. People were excluded if they had: (1) Upper limb movement deficits attributable to non-stroke pathology; and/or (2) Moderate to severe receptive aphasia (< 10 on “receptive skills” of Sheffield Screening Test for Acquired Language Disorders). Eight of the participants were male, mean age was 63.6 (SD = 11), mean time since stroke was 77 months (SD = 58). Participants underwent a structural scan, using a 3T (magnetom Skyra), Siemens Healthcare, T1-weighted image (TR, 5,000 ms; TE, 2.98 ms; field of view, 256 mm; matrix, 256_256; slice thickness, 1.0 mm, 176 slices) to identify the site of the lesion. Scans for 2 of the participants were unavailable due to contraindications (metal implant and pacemaker). Lesion locations for the remaining eight participants (Table 1) were identified visually from these scans by a trained radiographer and additionally confirmed by importing scans into mriCro, drawing around the lesion and superimposing an anatomical template (Automated Anatomical Labeling) onto the scan, which provided anatomical labels of the lesioned area.

**Table 1:** Characteristics of participants with stroke.

	**Handedness**	**Side of CVA**	**Previous CVA**	**Lesion site**	**Action research arm test**	**Wolf motor function test**	**Tardieu**	**Stroke impact scale**	**Nottingham sensory assessment**			**Star cancellation**	**Rey figure copy**	**McGill pain questionnaire**	**Mini-mental**
									**Touch**	**Proprioc-eption**	**2 pointdiscrimination**				
1	R	R	Y	Left pons	57	67	0	62	2	2	2	54	23	0	27
2	L	L	N	Left internal capsule extending into parietal cortex, left caudate, left putamen	41	60	2	63	2	2	2	54	35	0	27
3	L	L	N	Right periventricular region	56	71	0	78	2	2	1	54	33	0	21
4	R	L	N	Left parietal cortex near to falx, primary motor cortex, visual cortex (paracentral and precentral lobules)	57	75	0	78	2	2	1	54	31	0	29
5	R	L	N	Left parietal cortex, left mid and superior temporal lobe, left posterior limb internal capsule, left putamen	57	67	0	85	2	2	2	54	34	0	30
6	R	L	N	Right mid frontal lobe	54	67	0	74	2	2	2	54	29	1	28
7	L	R	N	Right parietal cortex and right cuneus	54	58	2	78	1^α^	2	2	52	30	0	29
8	R	L	N	Left lobe cerebellum	54	68	0	68	2	2	2	54	31	0	30
9	R	L	N	Unavailable	55	61	0	63	1	2	1^δ^	54	28	2	^§^
10	R	L	N	Unavailable	57	66	0	99	2	2	2	54	27	0	30

Action Research Arm Test−57 = maximum score; Wolf Motor Function Test–maximum score = 75; Stroke Impact Scale 100 = maximum score–mean score for all 8 domains is shown; Tardieu Scale–scores for elbow extension performed at medium velocity, 0, no resistance; 5, joint is immovable; Nottingham Sensory Assessment (Erasmus modification)−0, absent; 1, impaired; 2, normal; scores represent all joints (shoulder, elbow, wrist, fingers) unless otherwise indicated; Star cancellation−51-54 = normal; Rey figure copy−31-36 = normal; McGill−0, no pain; 5, excruciating; Mini-mental−25-30, normal;

α , impaired at wrist only;

δ , impaired and wrist and fingers only;

§ *, unable to test due to expressive dysphasia*.

The following assessments were used to characterize the group of stroke participants, with results displayed in Table 1. Physical tests to determine upper limb function were the Action Research Arm Test (35), the Wolf Motor Function Test (36); Nottingham Sensory Assessment [Erasmus MC modification (37)], Short-Form McGill Pain Questionnaire (38) and Tardieu Scale (39). To determine the participants cognition the Star Cancellation assessment, Sheffield Screening test for Acquired Language Disorders, Rey-Osterrieth Complex Figure (40), and the Mini Mental State Examination (41) were administered. The Stroke Impact Scale (42) was used to assess quality of life.

Ten healthy participants, matched to stroke participants on age, gender and hand dominance were also recruited from the Hunter Medical Research Institute Research Registry in Newcastle. All healthy participants' upper limb function was within normal limits of the Box and Block test (43).

### Research Protocol

#### Design

**Figure 1:**
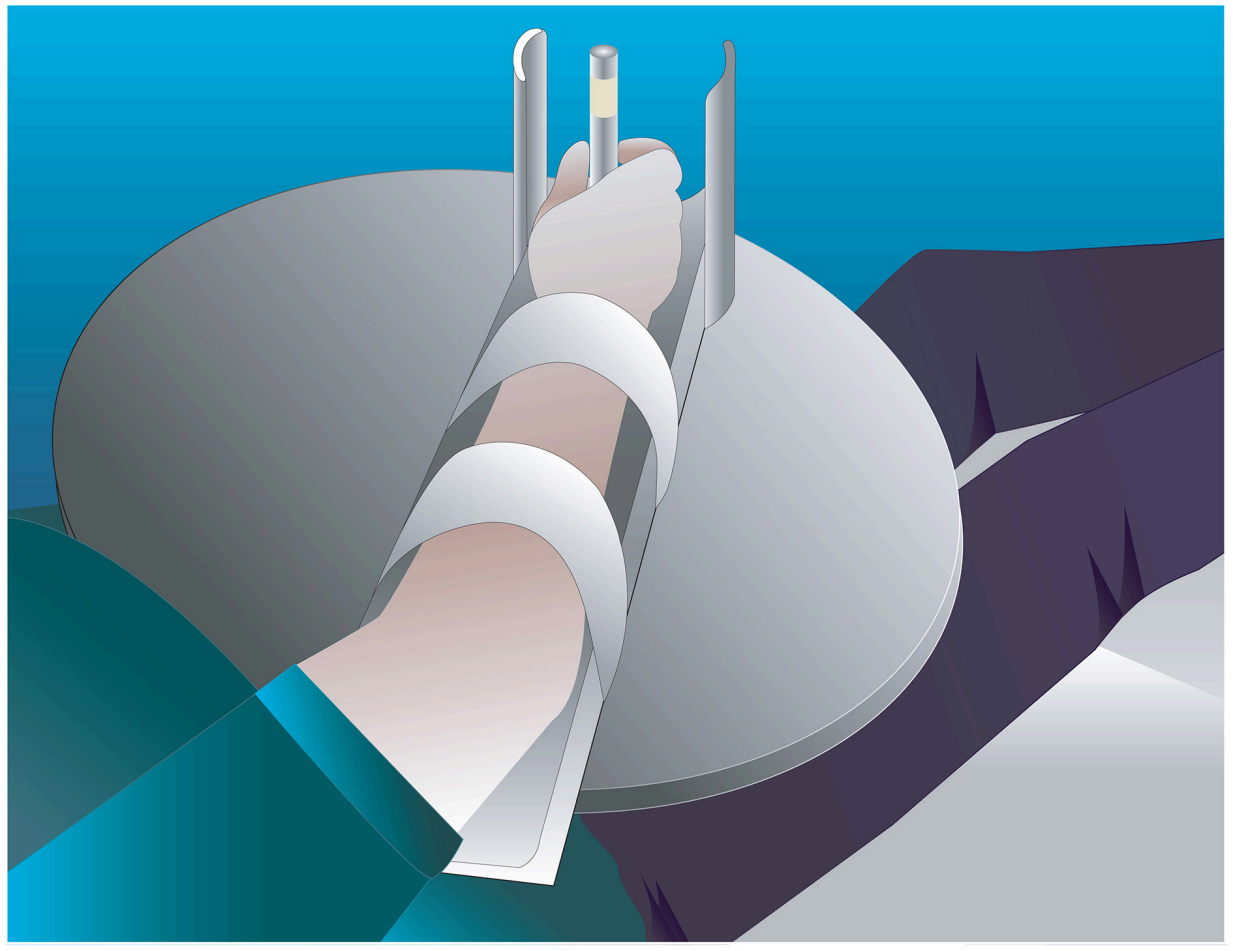
Task set-up for the splint condition.

Participants were required to perform two conditions; a standardized (using a splint) and a natural performance of a modified reach-to-grasp movement. The movement consisted of holding a cylindrical dowel, then opening the hand, flexing the elbow through a range of 20 degrees, and closing the hand on the cylindrical dowel again. Then the hand opened again, the elbow was extended 20 degrees and the hand was closed on the dowel again. Ten repetitions of the movement were performed in each condition. For both conditions the participants were supine on a 600 × 1,820 mm plinth, and the starting position was resting the mid-pronated forearm and hand in the base of the splint which measured 480 mm (h) × 85 mm (w) with a foam wedge to position the participant's upper limb into 20° shoulder flexion and 45° elbow flexion. The splint was attached with velcro to a curved edge lap desk (510 × 380 × 69 mm) that was positioned on the participant's trunk. The hand was visible by the participant throughout the movement. For the standardized condition, the forearm was secured to the splint with velcro strapping. The splint was made from lightweight plastic material, attached to a plastic base via a hinge joint set to lock at 20 degrees. A removable perspex rod 110 mm high and 15 mm diameter was attached to the base in one of two positions for right or left handed trials. Figure 1 shows the task set-up for the splint condition. In the natural condition, the midpronated forearm rested in the same splint base, but movement was performed without the perimeter limiting hand opening or the lock at 20° elbow flexion. Instead, the amount of elbow flexion was guided visually by the top of the rod, measuring a height of 210 mm, which when grasped, approximated 20° elbow flexion. Hand opening was guided by practice prior to data collection, where subjects copied the amount experienced in the splint with the perimeter, in the natural condition. Participants moved at their preferred speed. The order of conditions was randomized using a computerized random sequence. Each stroke participant performed a different random sequence. Healthy participants performed the same random sequence as their matched stroke participant, with the arm matching the side affected by the stroke participant, i.e., dominant or non-dominant.

Prior to data collection, the participant practiced at least four trials of each condition; two in the splint and two natural movements, with more practice if necessary. For both conditions the participants were instructed to “*Grasp the bottom marker of the rod using your thumb and finger tips, with your forearm resting in the splint (in the position demonstrated). When I say ‘start' open your hand whilst bending your elbow and grasp the top marker, then open your hand to return to the start position.”* The study was approved by Hunter New England Research Ethics Committee (Ref. No. 11/04/20/4.05). Informed written consent was obtained from all participants.

#### Data Acquisition and Analysis

Data were captured using a Qualisys™ 3D motion analysis system with three infrared cameras positioned in an arc near the foot of the plinth, at a sample rate of 200 Hz. The cameras recorded the movement of reflective markers attached to the hand and wrist, to capture the modified reach-to-grasp movement. Two spherical 7 mm reflective markers were fixed with double-sided tape to the lateral surface of the index finger (between the distal interphalangeal joint of the finger and the finger nail), and the medial surface of the thumb (between the distal interphalangeal joint of the thumb and the thumb nail) to measure grasp. A third 12 mm reflective marker was placed on the dorsal side of the forearm, on the radius, 7 cm proximal to the radial styloid process (to measure the transport phase) (44). The wrist marker was elevated by a 2 cm foam cube attached via a velcro strap to ensure visibility of the marker.

Data were processed using Qualisys Track Manager software and analyzed using custom Matlab programmes. Data were filtered using a low pass zero-phase Butterworth digital filter with a cut-off of 8 Hz. Trajectory, velocity, and acceleration were calculated from the three dimensional coordinates of each marker. Movement onset was defined as the time at which the 3D velocity exceeded 5% of the peak velocity value of the wrist (7). The transport component of the modified reach-to-grasp movement was described by the trajectory, velocity, and acceleration of the wrist marker (7). End of transport was defined as the first time at which the maximum distance of the wrist marker, in the combined x,y (horizontal) plane, was achieved. Distance was defined as the distance between the start and end point.

Grasp was described by the trajectory of the thumb and finger markers (7). The start of hand opening was defined as the time at which the planar (3-dimensional) between the thumb and finger marker exceeded 5% of the peak grasp aperture. Peak grasp aperture was defined as the maximum planar distance between the thumb and finger marker. The time to wrist peak velocity, wrist peak deceleration and peak aperture were determined and expressed in absolute and proportional (i.e., as a percentage of movement duration) terms.

#### Statistical Approach

Statistical analysis was conducted using the Statistica (Version 13.3) statistical software. To identify whether a relationship existed between the following kinematic parameters (a) start time of hand opening and start time of arm transport; (b) time of peak grasp aperture and time of peak deceleration; and (c) time of peak grasp aperture and time of peak velocity, Pearson's Product Moment Correlation Coefficients were calculated. Within-group correlation coefficients were calculated separately for each condition. Correlations were defined as strong (≥0.7), moderate (0.4–0.69), or low ( ≤ 0.39) (45).

To test the second and third hypotheses i.e., that the splint would cause significant changes to performance compared to movement without the splint, and that performance of the task by people with stroke would be significantly different to that of healthy individuals, differences in the means from the two groups (stroke and healthy) and the two conditions (natural and splint) were tested using multivariate analyses of variance (MANOVA) (46). Results were adjusted for multiple comparisons using least squares means of main effects and interaction effects. MANOVA was chosen instead of separate ANOVA tests for several reasons: (a) increased power—given the response variables are correlated, MANOVA can detect differences too small to be detected through individual ANOVAs; (b) MANOVA can detect multivariate response patterns: the factors [condition, group (stroke vs. healthy)] may influence the relationship between responses (dependent variables) rather than affecting a single response. Single-response ANOVAs can miss these multivariate patterns; (c) MANOVA reduces the likelihood of Type I error: the chance of incorrectly rejecting the null hypothesis increases with each successive ANOVA, whereas running one MANOVA to test all response variables simultaneously keeps the family error rate equal to the alpha level.

As mentioned earlier, movement duration is a key control parameter in reach-to-grasp, with the reach being planned with respect to the expected movement duration to the target. To give a more complete understanding of how these kinematic variables reflect the effort to ensure that the expected duration to the target of each of transport and grasp are temporally matched within an overall model for control of reach to grasp, additional analyses were conducted using ANCOVA (46) to determine how the multiple kinematic variables covaried with movement duration as the independent outcome variable.

To further explore how the multiple kinematic variables covaried with each other, and whether variables behaving similarly could be grouped into a smaller number of construct variables, a factor analysis was performed (46). Then, the same MANOVA and ANCOVA analyses were performed on the factors.

## Results

### Relationship Between Grasp and Transport at the Start of the Reach

The start of transport of the hand and start of grasp aperture were significantly correlated in all conditions for both groups. Correlations were low for the stroke group: Stroke/natural (*r* = 0.234, *p* = 0.037); Stroke/splint (*r* = 0.275, *p* = 0.015); and strong for the healthy group: Healthy/natural (*r* = 0.864, *p* = 0.000); Healthy/splint (*r* = 0.822, *p* = 0.000).

### Relationship Between Grasp and Transport at the Time of Peak Grasp Aperture

In three of the conditions (stroke/natural, healthy natural, and healthy/splint) the group mean absolute time of peak velocity occurred first, followed by the mean absolute peak aperture time and then the mean absolute time of peak deceleration (Table 2). In the stroke/splint condition, mean absolute peak aperture time occurred first, followed by the mean absolute time of peak velocity, then the mean absolute time of peak deceleration. The mean percentage times of these kinematic parameters follow the same pattern as the absolute times, except for the healthy natural condition, where the % peak aperture preceded % peak velocity.

**Table 2:** Means and standard error of kinematic variables.

	**Stroke/natural**		**Stroke/splint**		**Healthy/natural**		**Healthy/splint**	
	**Mean**	**(SE)**	**Mean**	**(SE)**	**Mean**	**(SE)**	**Mean**	**(SE)**
Movement duration (ms)	1,412	(44)	1,278	(48)	937	(30)	918	(34)
Distance moved (mm)	58	(2)	76	(3)	44	(2)	54	(3)
**TRANSPORT COMPONENT**								
Start time of transport^*^ (ms)	740	(48)	945	(50)	509	(32)	800	(37)
Peak velocity (mm/s)	101	(3)	121	(4)	119	(4)	172	(6)
Time to peak velocity (ms)	491	(27)	572	(24)	361	(19)	430	(19)
Time to peak velocity (%)	35	(2)	46	(2)	39	(2)	48	(2)
Time to peak deceleration (ms)	662	(31)	755	(26)	535	(20)	575	(25)
Time to peak deceleration (%)	48	(2)	62	(2)	58	(2)	63	(2)
**GRASP COMPONENT**								
Start time of aperture (ms)	882	(94)	740	(46)	490	(31)	512	(41)
Peak aperture size (mm)	96	(2)	85	(2)	105	(2)	100	(2)
Time to peak aperture (ms)	609	(41)	552	(40)	330	(21)	442	(31)
Time of peak aperture (%)	43	(3)	42	(3)	36	(2)	49	(3)

* *Start time of transport and start time of aperture represent time elapsed (in ms) since the start signal was given. All other times represent time since movement onset*.

The absolute time of peak velocity and the absolute time of peak grasp aperture were significantly and positively correlated in all conditions for both groups. Correlations ranged from low to moderate in the stroke group: Stroke/natural (*r* = 0.272, *p* = 0.015); Stroke/splint (*r* = 0.495, *p* = 0.000); and were moderate in the healthy group: Healthy/natural (*r* = 0.510, *p* = 0.000); Healthy/splint (*r* = 0.573, *p* = 0.000).

The absolute time of peak deceleration and the absolute time of peak grasp aperture were significantly and positively correlated in both conditions in the healthy group with a moderate level of correlation: Healthy/natural (*r* = 0.422, *p* = 0.000); Healthy/splint (*r* = 0.625, *p* = 0.000). This relationship was not significant for the stroke group in the splint condition (*r* = 0.076, *p* = 0.509), or the natural condition (*r* = 0.002, *p* = 0.984).

### Effect of Splint and Group

#### MANOVA on Kinematic Variables

Means and standard deviation for all kinematic variables are shown in Table 2. Results for MANOVA of group, condition and their interaction modeling all variables together shows a highly significant Wilks Lambda statistic for group (**Λ** = 0.547 [*F*_(11, 290)_ = 21.8, *p* = 0.000], condition (**Λ** = 0.608, [*F*_(11, 290)_ = 17, *p* = 0.000] and group by condition interaction (**Λ**0.821 [*F*_(11, 290)_ = 5.8, *p* = 0.000]). At the univariate level, the group comparison showed a significant difference between the stroke group and the healthy group for all variables except for percentage peak aperture time. Thus, for the stroke group, movement duration was longer [*F*_(1, 300)_ = 73.98; *p* < 0.0001], peak velocity was smaller [*F*_(1, 300)_ = 63.3; *p* < 0.0001], peak aperture was smaller [*F*_(1, 300)_ = 21.5; *p* < 0.0001], and the absolute times of peak velocity [*F*_(1, 300)_ = 35.2; *p* < 0.0001], peak deceleration [*F*_(1, 300)_ = 34.0; *p* < 0.0001] and peak aperture were later [*F*_(1, 300)_ = 30.9; *p* < 0.0001], and percentage time of peak velocity [*F*_(1, 300)_ = 3.9; *p* = 0.048] and percentage time of peak deceleration [*F*_(1, 300)_ = 8.68; *p* = 0.004] were earlier (Table 2).

The univariate comparisons for condition showed significant differences for all variables except movement duration and absolute time of peak aperture. Thus, for the splint condition, peak velocity size was higher [*F*_(1, 300)_ = 73.9; *p* < 0.0001], absolute time of peak velocity [*F*_(1, 300)_ = 10.6; *p* < 0.0001], and peak deceleration were later [*F*_(1, 300)_ = 6.4; *p* = 0.012], percentage time of peak velocity [*F*_(1, 300)_ = 37.0; *p* < 0.0001], peak deceleration [*F*_(1, 300)_ = 25.8; *p* < 0.0001], and peak aperture [*F*_(1, 300)_ = 5.4; *p* = 0.021] were later and peak aperture was smaller [*F*_(1, 300)_ = 9.6; *p* = 0.002] (Table 2).

There were significant group by condition interactions for the following variables: peak velocity size [*F*_(1, 300)_ = 15.8; *p* < 0.0001], percentage peak deceleration time [*F*_(1, 300)_ = 6.9; *p* = 0.009], absolute peak aperture time [*F*_(1, 300)_ = 5.9; *p* = 0.017], and percentage peak aperture time [*F*_(1, 300)_ = 6.75; *p* = 0.010]. Thus, the increased velocity size and the later absolute and percentage peak aperture time observed with the splint, were most evident in the healthy group, compared to the stroke group (Table 2). The later percentage peak deceleration time in the splint condition however, was more prevalent in the stroke group than the healthy group.

#### MANOVA on Factors

##### Factor analyses

Factor analysis showed that overall, the kinematic variables could be grouped into four factors (Table 3) which accounted for 70% of the variance. An eigenvalue of >1 was used to justify the factors. Each factor contained linear relationships of two or more variables. Factor 1 consisted of the following variables for the timing of hand transport: time to peak velocity, percentage time to peak velocity, time to peak deceleration, and percentage time to peak deceleration. Factor 2 was comprised of the variables for timing of grasp: time to peak aperture and percentage time to peak aperture. Factor 3 related to speed of transport and aperture size: size of peak velocity and size of peak aperture, and factor 4 was a combination of the start time of each of transport and grasp, along with the distance moved by the wrist. For ease of description, the factors will be labeled as follows: Factor 1 = “Timing of transport;” Factor 2 = “Timing of grasp;” Factor 3 = “Speed and aperture size;” and Factor 4 = “Getting started and distance moved.” “Timing of transport” was the most influential, accounting for 26% of the variance, followed by “Timing of grasp,” accounting for 19%.

**Table 3:** Factor loadings (varimax normalized).

	**Factor 1 “Timing of transport component”**	**Factor 2 “Timing of grasp component”**	**Factor 3 “Speed and aperture size”**	**Factor 4 “Getting started and distance moved”**
Time to peak velocity (ms)	**0.719**	0.357	0.278	0.216
Time to peak velocity (%)	**0.800**	0.148	−0.369	0.098
Peak velocity (mm.s^−1^)	−0.056	0.147	**0.833**	−0.231
Time to peak deceleration (ms)	**0.782**	0.062	0.463	0.066
Time to peak deceleration (%)	**0.797**	−0.195	−0.198	−0.118
Time to peak aperture (ms)	0.060	**0.929**	0.241	0.129
Time of peak aperture (%)	0.067	**0.904**	−0.220	0.033
Peak aperture size (mm)	0.023	−0.097	**0.549**	0.149
Start time of transport (ms)	0.041	0.032	0.047	**0.820**
Start time of aperture (ms)	−0.172	0.203	0.371	**0.543**
Distance moved (mm)	0.149	0.031	−0.159	**0.708**
Eigenvalue	2.816	2.056	1.544	1.369
% total variance	25.6	18.7	14	7.8

*Oblique factors, clusters of significant loadings are shown in bold type*.

Comparison of factors between groups and conditions were performed using MANOVAs. Results for group, condition and their interaction modeling all factors together shows a highly significant Wilks Lambda statistic for group (**Λ** = 0.636, [*F*_(4, 297)_ = 42.6, *p* = 0.000]), condition (**Λ** = 0.744, [*F*_(4, 297)_ = 25.6, *p* = 0.000]) and group by condition interaction (**Λ** = 0.950 [*F*_(4, 297)_ = 3.9, *p* = 0.004]).

At the univariate level, the group comparison of factors showed a significant difference between the stroke group and the healthy group for “Timing of grasp” [*F*_(1, 300)_ = 4.94, *p* = 0.027], “Speed and aperture size” [*F*_(1, 300)_ = 71.8, *p* = 0.000], and “Getting started and distance moved” (*F* (1, 300) = 64.3, *p* = 0.000), but at 10% significance for “Timing of transport” [*F*_(1, 300)_ = 3.80, *p* = 0.052]. Thus, for the stroke group, the “timing of grasp component” was later, “speed and aperture size” were smaller, and “Getting started and distance moved” was later.

The univariate comparisons for condition showed significant differences for factors “Timing of transport” [*F*_(1, 300)_ = 26.5, *p* = 0.000], “Speed and aperture size” [*F*_(1, 300)_ = 36.4, *p* = 0.000]; and “Getting started and distance moved” [*F*_(1, 300)_ = 34.7, *p* = 0.000], but not for “Timing of grasp.” Thus, for the splint condition, “Timing of transport component” was later, “Speed and aperture size” were larger and values for “Getting started and distance moved” were later.

There were significant group by condition interactions for “Timing of transport” [*F*_(1, 300)_ = 4.05, *p* = 0.045], “Timing of grasp” [*F*_(1, 300)_ = 8.56, *p* = 0.004], and “Speed and aperture size” [*F*_(1, 300)_ = 3.88, *p* = 0.050, significant at 10%], but not for “Getting started and distance moved.” So for “timing of transport,” the splint caused later transport values in the stroke group compared to the healthy group; for “timing of grasp,” the splint caused earlier values for the stroke group but later values for the healthy group; and the increase in “speed and aperture size” were smaller for the stroke group compared to the healthy group. Table 4 shows a summary of significant findings for both kinematic variables and factors from the MANOVAs.

**Table 4:** Summary of significant findings for both kinematic variables and factors, showing effect of group, condition, and interaction.

**Kinematic parameter/factor**	**Group**	**Condition**	**Interaction by group and condition**
Time to peak velocity (ms)	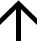	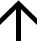	NS
Time to peak velocity %	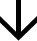	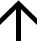	NS
Peak velocity	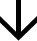	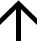	Increased more in healthy group (splint condition)
Time to peak deceleration (ms)	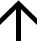	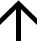	NS
Time to peak deceleration %	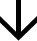	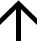	Increased more in stroke group (splint condition)
Time to peak aperture (ms)	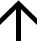	NS	Increased more in healthy group (splint condition)
Time of peak aperture %	NS	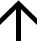	Increased more in healthy group (splint condition)
Peak aperture size	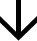	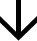	NS
Movement duration	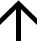	NS	NS
Factor 1 timing of transport	NS	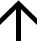	Increased more in stroke group than healthy group (splint condition)
Factor 2 timing of grasp	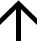	NS	Decreased in stroke group (splint condition) but increased in healthy group (splint condition)
Factor 3 speed and aperture size	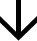	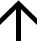	Increased more in healthy group than stroke group (splint condition)
Factor 4 getting started and distance moved	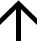	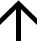	NS

*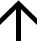, kinematic parameter was larger compared to control group/no splint condition; 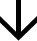, kinematic parameter was smaller compared to control group/no splint condition; NS, not significant*.

### Covariation of Kinematic Variables and Factors With Movement Duration

#### Covariation of Kinematic Variables

A MANCOVA analysis was done to determine the influence of the kinematic variables on movement duration, in different groups and conditions (Table 5). A *later absolute* time to peak velocity and peak deceleration in all groups and conditions, and absolute time to peak aperture in only the stroke group (both conditions) was predictive of a longer movement duration. However, an *earlier percentage* time to peak velocity and peak deceleration in all conditions and percentage time to peak aperture in only the stroke group (both conditions) was predictive of a longer movement duration. A smaller size of peak velocity was predictive of a longer movement duration, only in the stroke group. Peak aperture size and distance moved were not predictive of movement duration in any condition or group. In the stroke, natural condition, a later start time of transport was predictive of a shorter movement duration whereas a later start time of aperture was predictive of a longer movement duration.

**Table 5:** ANCOVA parameter estimates and *p-*values for each group and condition for kinematic variables (parameter estimates show increase or decrease in comparison to the intercept, thus a positive value indicates a positive correlation of the variable with movement duration, and “–” indicates a negative correlation with movement duration).

**Kinematic variable**	**Condition**	**Estimate**	**Standard error**	**95% LCL**	**95% UCL**	***P-*value**
Absolute time to peak velocity	Stroke, natural	0.383	0.118	0.1524	0.6136	0.0011
	Stroke splint	0.460	0.101	0.2627	0.6567	0.0000
	Healthy, natural	0.783	0.395	0.0084	1.5569	0.0476
	Healthy, splint	1.209	0.343	0.5367	1.8820	0.0004
Percentage time to peak velocity	Stroke, natural	−0.0051	0.0019	−0.0088	−0.0014	0.0071
	Stroke splint	−0.058	0.0016	−0.0088	−0.0027	0.0002
	Healthy, natural	−0.0073	0.0045	−0.0161	0.0015	NS
	Healthy, splint	−0.0125	0.0040	−0.0203	−0.0047	0.0016
Peak velocity	Stroke, natural	−0.00086	0.000258	−0.0014	−0.0004	0.0008
	Stroke splint	−0.00097	0.000325	−0.0016	−0.0003	0.0029
	Healthy, natural	−0.00050	0.000409	−0.0013	0.0003	NS
	Healthy, splint	0.00018	0.000264	−0.0005	0.0005	NS
Absolute time to peak deceleration	Stroke, natural	0.4992	0.0764	0.3500	0.6489	0.0000
	Stroke splint	0.5985	0.0767	0.4482	0.7488	0.0000
	Healthy, natural	1.1718	0.2995	0.5837	1.7578	0.0001
	Healthy, splint	0.6777	0.3160	0.0586	1.2969	0.0319
Percentage time to peak deceleration	Stroke, natural	−0.0089	0.0012	−0.0114	−0.0065	0.0000
	Stroke splint	−0.0108	0.0013	−0.0134	−0.0082	0.0000
	Healthy, natural	−0.0113	0.0032	−0.0175	−0.0051	0.0003
	Healthy, splint	−0.0063	0.0036	−0.0133	0.0075	0.0796
Absolute time to peak aperture	Stroke, natural	0.5042	0.0494	0.40733	0.60114	0.0000
	Stroke splint	0.1422	0.0708	0.00351	0.2810	0.0441
	Healthy, natural	0.2332	0.2217	−0.20133	0.6676	NS
	Healthy, splint	−0.2283	0.1813	−0.58373	0.1270	NS
Percentage time to peak aperture	Stroke, natural	−0.0084	0.0008	−0.0101	−0.0068	0.0000
	Stroke splint	−0.0022	0.0010	−0.0042	−0.00014	0.0361
	Healthy, natural	−0.0030	0.0025	−0.0079	0.00182	NS
	Healthy, splint	0.0021	0.0017	−0.00129	0.00554	NS
Peak aperture	Stroke, natural	0.00028	0.00034	−0.00039	0.00094	NS
	Stroke splint	−0.00035	0.00039	−0.00110	0.00041	NS
	Healthy, natural	0.0008	0.00047	−0.00012	0.00017	0.0874
	Healthy, splint	−0.00018	0.00151	−0.00118	0.00082	NS
Start time traNSport	Stroke, natural	−0.0387	0.0162	−0.07042	−0.0070	0.0167
	Stroke splint	−0.0137	0.0220	−0.05721	0.0298	NS
	Healthy, natural	0.0344	0.0750	−0.11264	0.1815	NS
	Healthy, splint	0.0038	0.0678	−0.12917	0.1367	NS
Start time aperture	Stroke, natural	0.02348	0.0069	0.0099	0.0871	0.0007
	Stroke splint	0.00032	0.0147	−0.0286	0.0292	NS
	Healthy, natural	0.0552	0.0808	−0.1031	0.2136	NS
	Healthy, splint	−0.020	0.0727	−0.1622	0.1228	NS
Distance moved	Stroke, natural	0.000630	0.000458	−0.00027	0.00153	NS
	Stroke splint	0.000260	0.000462	−0.00065	0.00117	NS
	Healthy, natural	0.000293	0.0005	−0.00069	0.001275	NS
	Healthy, splint	0.000183	0.00049	−0.00077	0.001138	NS

NS denotes “not significant.”

#### Covariation of Factors

A later “timing of transport” in the stroke group was predictive of a longer movement duration in the natural condition (Table 6). A later “timing of grasp” was predictive of a longer movement duration in both groups for the natural condition, but just in the stroke group for the splint condition. A greater “speed and aperture size” was predictive of a shorter movement duration in all groups and conditions. Finally, a later/greater “getting started and distance moved” was predictive of a longer movement duration in all groups and conditions.

**Table 6:** ANCOVA parameter estimates and *p*-values for each group and condition for FACTORS (parameter estimates show increase or decrease in comparison to the intercept, thus a positive value indicates a positive correlation of the factor with movement duration, and “–” indicates a negative correlation with movement duration).

**Factor**	**Condition**	**Estimate**	**Standard error**	***P*-value**
1	Stroke, natural	0.072	0.014	0.0000
	Stroke splint	−0.023	0.019	NS
	Healthy, natural	0.062	0.035	0.0765
	Healthy, splint	−0.001	0.042	NS
2	Stroke, natural	0.066	0.017	0.0001
	Stroke splint	0.111	0.016	0.0000
	Healthy, natural	0.144	0.042	0.0007
	Healthy, splint	0.064	0.035	0.0695
3	Stroke, natural	−0.230	0.021	0.0000
	Stroke splint	−0.272	0.021	0.0000
	Healthy, natural	−0.234	0.034	0.0000
	Healthy, splint	−0.225	0.028	0.0000
4	Stroke, natural	0.053	0.018	0.0031
	Stroke splint	0.056	0.032	0.0030
	Healthy, natural	0.097	0.041	0.0028
	Healthy, splint	0.113	0.033	0.0055

NS denotes “not significant.”

## Discussion

The timing of key kinematic parameters in the performance of the modified reach-to-grasp task was such that the median peak velocity occurred within 50% of movement duration (Table 2) and during the movement, the peak velocity occurred first, followed by peak aperture and then peak deceleration in three out of the four conditions.

The results also demonstrate that the temporal coupling between key grasp and transport events are present in this modified reach-to-grasp task, in healthy people and in the stroke group, with the exception of the correlation between time of peak aperture and time of peak deceleration. Correlations ranged from moderate to strong in the healthy group and from low to moderate in the stroke group.

### Effect of the Splint

Wearing the splint did cause some differences in the kinematics of the movement compared to the more natural performance, as hypothesized. Peak velocity was higher, absolute and percentage time of peak velocity and peak deceleration were later, percentage time of peak aperture was later, and peak aperture size was smaller, when wearing the splint.

The later timing of the peak velocity and peak deceleration implies that the splint caused a more ballistic mode of control, with less use of feedback during a shortened deceleration phase, and more reliance on the generation of centralized motor commands prior to movement execution. This effect was more evident in the stroke group with regard to percentage time of peak deceleration. As people with stroke have an abnormally long deceleration phase, wearing the splint appears to have actually normalized their movement in this respect.

The later percentage peak aperture time follows the later times of the transport events, which likely reflects a preservation of the invariant temporal relationship between transport and grasp that ensures a coordinated reach-to-grasp. Stroke participants were less able to make this adjustment of aperture timing (Table 4), which concurs with previous research showing weaker correlation of transport and grasp events in people with stroke (7, 14).

The splint also caused a further distance moved by the wrist (Table 2). This could account for the higher peak velocity and later absolute times of peak velocity and peak deceleration, which may have been required to generate enough force to cover the larger distance. This effect was demonstrated less by the stroke group, who had a smaller increase in wrist distance and peak velocity than in healthy participants, when wearing the splint. This is understandable, as people with stroke have difficulty in generating force to adjust movement extent (47).

The MANOVA on the factors followed the findings from the MANOVA on the kinematic variables. The splint led to later timing of transport events and a later timing of grasp as a proportion of movement duration. The smaller peak aperture found with the splint condition did not appear in the factor “speed and aperture size,” and this could be because the increase in speed outweighed the relatively smaller decrease in aperture size.

Therefore, the natural condition is a more appropriate task to use when performing the modified reach-to-grasp task than the splint condition, because the splint significantly alters the kinematic measures that reflect motor control.

### Differences Between the Healthy and Stroke Group

Our third hypothesis concerned whether the performance of the task by people with stroke would be significantly different to that of healthy individuals.

The stroke group showed a smaller aperture (18), a longer movement duration (15) with a corresponding smaller peak velocity (15) and an earlier percentage time of peak velocity and peak deceleration (7), indicating a longer deceleration phase. The longer deceleration phase allows more time to use visual and proprioceptive feedback mechanisms to guide the movement, supposedly to compensate for altered generation of motor commands from damaged neural networks (4). The absolute times to reach peak velocity, peak aperture, and peak deceleration were longer in the stroke group, reflecting the greater wrist distances that were moved by stroke participants (Table 2). Similarly, previous studies demonstrated that people with stroke show errors in movement extent (47) and tend to overshoot close targets (21).

In general, the MANOVA on the factors followed the findings from the MANOVA on the kinematic variables. That is, stroke caused a smaller speed and aperture size, later timing of grasp, and a larger distance moved and later start time. Therefore, in future studies it might be possible to use the factors instead of all the individual kinematic variables in the statistical analyses.

### Covariation of Dependent Variables With Movement Duration: Insights Into Overall Motor Control

Because of the importance of movement duration as a determining parameter in controlling reach-to-grasp, we analyzed how the dependent variables and the factors derived from these, related to movement duration. Interestingly, kinematic variables relating to timing of transport (and the “timing of transport” factor) were predictive of movement duration in both groups, whereas kinematic variables relating to timing of grasp were only predictive of movement duration in the stroke group. The stronger relationship between grasp and movement duration in the stroke group could be interpreted as indicating that grasp events mirrored those of transport more often in stroke participants, suggesting they were more dependent on this coordination between grasp and transport, to accomplish the task, than the healthy group. This relationship is also demonstrated by a later start time of aperture predicting a longer movement duration, in the stroke, natural condition, but not in the healthy group. This likely reflects the difficulty people with stroke have in generating force in finger extensors to open the hand, so they need longer to accomplish this. Studies of the kinematics of reach-to-grasp rarely report measures of start time of hand aperture, so the finding that this aspect was influential in planning movement duration in a reach-to-grasp task is interesting.

This adaptation of movement duration to a later start time of aperture also implies a preservation of the ability to adapt movement duration to compensate for deficits in aspects of the reach-to-grasp in the stroke group. This preservation is also reflected in the fact that a later start time of transport was predictive of a shorter movement duration, in the stroke, natural condition, which was presumably compensatory for a delayed start time.

Peak aperture size was not an important determining factor for movement duration in either group or condition, and neither was the distance moved. Although the factors containing peak aperture and distance covaried with movement duration, this was caused by other elements within the factors—the size of peak velocity and start time.

### Comparison to Kinematics of Seated Reach to Grasp Tasks

Finally, it is interesting to note whether the timing of key kinematic parameters in the performance of the modified reach-to-grasp task follows a similar movement organization to the more commonly reported seated reach-to-grasp tasks. As in previous studies [see (4) for a review], the median peak velocity occurs within 50% of movement duration (Table 2) and during the movement, the peak velocity occurs first, followed by peak aperture and then peak deceleration in three out of the four conditions.

The results also demonstrate that the temporal coupling between key grasp and transport events reported in the literature for seated reach-to-grasp movements, are also present in this modified reach-to-grasp task, in healthy people and for the most part in the stroke group, with the exception of the correlation between time of peak aperture and time of peak deceleration. Correlations ranged from moderate to strong in the healthy group and from low to moderate in the stroke group. Thus, in this respect, the modified reach-to-grasp movement is similar to seated reach-to-grasp movements (7, 8) and is similarly indicative of a coordinated motor schema for reach-to-grasp in the brain (5).

As with previous studies comparing seated reach-to-grasp in people with stroke to healthy performance, the stroke group showed a smaller aperture (18), a longer movement duration (15) with a corresponding smaller peak velocity (15) and an earlier percentage time of peak velocity and peak deceleration (7), indicating a longer deceleration phase.

The findings regarding covariation of dependent variables with movement duration are also in agreement with studies of seated reach-to-grasp in healthy subjects, where timing of transport events are frequently associated with movement duration (9, 48). Our finding that peak aperture size was unimportant in relation to movement duration, however, is in contrast to a previous study investigating potential algorithms used by the central nervous system to control seated reach-to-grasp (49) which found that peak velocity size, peak aperture size and hand acceleration were all used to regulate reach-to-grasp within a model determining hand closure distance.

Because these findings are in common with studies with seated reach-to-grasp tasks, it can be concluded that it is possible to demonstrate the expected differences between stroke and healthy populations using this modified reach-to-grasp task.

## Study Limitations

The participants with stroke included in this study had mild to moderate arm motor impairment. Therefore, it is not yet known whether the results can be generalized to people with more severe deficits. The suitability of the modified reach-to-grasp task should be evaluated in people with more severe impairment in future research. Our method did not shed any light on muscle activation occurring during reach-to-grasp in supine, which one would expect to be adjusted for the altered relationship with gravity, compared to the seated position.

## Conclusions

Overall the modified reach-to-grasp task performed in supine shows similar kinematic characteristics to seated reach-to-grasp tasks and is therefore a method that could be used in the constraints of the fMRI environment to explore and better understand coordination and motor control of reach-to-grasp in people with stroke. Such research is sorely needed (50) and will increase knowledge about the neurobiological basis of recovery to inform the discovery of new treatments to improve motor control. Our recommendation is to have participants perform the movement without the splint, since wearing it caused differences in key kinematic variables compared to the natural condition. Instead, we suggest that participants receive sufficient training of the task beforehand, to encourage consistent performance, and that their performance is recorded with the use of motion capture systems compatible with the MRI environment.

This task may also have applications outside of the fMRI environment. Many people in the stroke population do not have sufficient generation of force in their arm muscles to perform reach-to-grasp movement in a sitting position, where the whole arm must move against gravity. Therefore, the modified reach-to-grasp movement could potentially enable reach-to-grasp coordination and motor control to be assessed in a wider number of people, earlier in the recovery process than previously.

## Ethics Statement

The study was approved by Hunter New England Research Ethics Committee (Ref. No. 11/04/20/4.05). Informed written consent was obtained from all participants.

## Author Contributions

PvV designed the study, supervised the work and led the writing of this manuscript. KB conducted the study as part of her Honours Bachelor in Occupational Therapy degree, assisted by fellow Honours Bachelor in Occupational Therapy student KP, and research assistant AD. IH led and conducted the statistical analyses. JA and TZ advised on MRI scans. HT identified lesion sites. JK and GC provided computer algorithms for data analysis.

### Conflict of Interest Statement

The authors declare that the research was conducted in the absence of any commercial or financial relationships that could be construed as a potential conflict of interest.
